# Approaches to Next-Generation Capripoxvirus and Monkeypox Virus Vaccines

**DOI:** 10.3390/v17020186

**Published:** 2025-01-27

**Authors:** Anna-Lise Williamson

**Affiliations:** 1 Institute of Infectious Disease and Molecular Medicine, University of Cape Town, Observatory, Cape Town 7925, South Africa; anna-lise.williamson@uct.ac.za; 2 Division of Medical Virology, Department of Pathology, Faculty of Health Sciences, University of Cape Town, Observatory, Cape Town 7925, South Africa

**Keywords:** poxvirus, vaccines, LSDV, mpox: lumpy skin disease

## Abstract

Globally, there are two major poxvirus outbreaks: mpox, caused by the monkeypox virus, and lumpy skin disease, caused by the lumpy skin disease virus. While vaccines for both diseases exist, there is a need for improved vaccines. The original vaccines used to eradicate smallpox, which also protect from the disease now known as mpox, are no longer acceptable. This is mainly due to the risk of serious adverse events, particularly in HIV-positive people. The next-generation vaccine for mpox prevention is modified vaccinia Ankara, which does not complete the viral replication cycle in humans and, therefore, has a better safety profile. However, two modified vaccinia Ankara immunizations are needed to give good but often incomplete protection, and there are indications that the immune response will wane over time. A better vaccine that induces a long-lived response with only one immunization is desirable. Another recently available smallpox vaccine is LC16m8. While LC16m8 contains replicating vaccinia virus, it is a more attenuated vaccine than the original vaccines and has limited side effects. The commonly used lumpy skin disease vaccines are based on attenuated lumpy skin disease virus. However, an inactivated or non-infectious vaccine is desirable as the disease spreads into new territories. This article reviews novel vaccine approaches, including mRNA and subunit vaccines, to protect from poxvirus infection.

## 1. Background to Mpox and Lumpy Skin Disease

Poxviruses are a family of large-DNA viruses divided into two subfamilies: the *Chordopoxvirinae* that infect vertebrates and the *Entomopoxvirinae* that infect insects. While there are 18 genera in *Chordopoxvirinae,* this review will concentrate on two genera, namely the Orthopoxviruses and the Capripoxviruses. There have been two notable outbreaks of poxvirus infections in recent years. The first is lumpy skin disease, mainly in cattle, caused by the Capripoxvirus lumpy skin disease virus (LSDV). The other is the Orthopoxvirus, monkeypox virus (MPVX), which causes mpox in humans and is closely related to the virus that causes smallpox. Mpox in humans is considered a zoonosis and probably originated in rats or squirrels [1].

The last case of natural smallpox was recorded in 1977 after an intensive global vaccination campaign [2]. Smallpox vaccination also protects against other zoonotic Orthopoxvirus infections, including monkeypox virus (MPXV), cowpox virus, camelpox virus, and vaccinia virus (VACV) [3]. The last smallpox vaccinations in Africa were administered around 1980. This means that a younger, unvaccinated population is now susceptible to MPXV infection. While mpox has been endemic in parts of West and Central Africa, it was only when it spread to other continents that there was a global response to the outbreaks. In July 2022, mpox outbreaks were declared a public health emergency of international concern as there were approximately 102,997 cases and 223 deaths across six World Health Organization (WHO) regions. Last year (2024), the Africa Centres for Disease Control and Prevention and the WHO declared the latest outbreak of mpox in Africa a public health emergency. An epidemic in the Democratic Republic of Congo has spread to neighboring countries, and this year, as of September 2024, there have been 32,010 reported mpox cases in 15 African nations [4].

There are two clades of MPXV described. Clade 1, which is endemic in Central Africa, has the highest severity of mpox. A new MPXV lineage was reported, and so clade I is now divided into clade IA and IB. Clade IA has a higher mortality than clade IB [5]. Clade IB is spreading from DR Congo through central Africa and has been reported in Uganda and Rwanda. The mpox deaths in Africa caused by clade 1B are seen mainly in children (78%). Mpox has also been reported to cause stillbirths in the region [6]. In West Africa, clade II occurs in less severe cases than observed with clade 1 in Central Africa. Until recently [7], all the human cases outside of Africa have been due to MPXV clades IIa and IIb [8]. The smallpox vaccine can protect against both strains of MPXV, clades I and II. MPXV genomes were found to be 99% identical within geographic regions and 95% similar across different geographical regions [9,10]. The structural proteins of MPXV are well conserved, with most having over 95% identity to VACV proteins [11].

As a notifiable disease, LSD is of economic significance. In cattle, LSDV infection can cause decreased milk and meat production and significant damage to hides [12,13]. LSD was initially reported in Zambia in 1929 and confined to Africa until 1989, when it spread from Africa to Israel [7] and then to other countries in the Middle East [14]. This century, LSDV spread to the Balkans (2015) and Serbia (2016) [15]. LSD has now been reported in countries in Europe and Asia, including Bangladesh, Sri Lanka, Mongolia, China, India, Pakistan, Thailand, Nepal, Indonesia, Vietnam, and Russia [16,17,18,19,20,21,22,23,24]. The outbreak in the Balkans was controlled by vaccination with a live attenuated LSD vaccine based on the Neethling strain. Outbreaks in parts of Europe have been controlled [25], but LSD will likely be endemic in most of Africa and Asia. Vaccination is the best way to prevent the spread of LSDV, as subclinical infections are common, and therefore, identification of infected animals is not always possible [26].

## 2. Background on Poxviruses Relevant to Vaccine Design

Poxviruses are large and complex DNA viruses coding for between 120 and 300 proteins [27]. MPXV has a genome size of ~197 kbp comprising 190 open reading frames, and LSDV has a genome size around ~150 kbp and codes for 156 putative proteins [11,28,29]. The replication cycle of the poxvirus is complex, with the virus replicating in the cytoplasm. Poxviruses are relatively stable viruses as they have a proofreading DNA polymerase, which limits their mutation rate compared to other viruses [8]. However, the poxviruses have other mechanisms for evolving besides point mutations. These include recombination with host gene homologues [30] as well as other poxviruses [31,32]. Horizontal gene transfer from the host to the poxvirus enables the virus to respond to the host’s defense mechanisms [33]. Many poxviruses have extensive gene duplication of these genes, resulting in paralogous gene families [34]. Extensive recombination between LSDV and other Capripoxviruses has been reported in Asia. As many recombination events were detected in one of the vaccine stocks, the recombinants likely resulted from vaccine manufacturing mismanagement rather than recombination events in the field [14].

**Figure 1:**
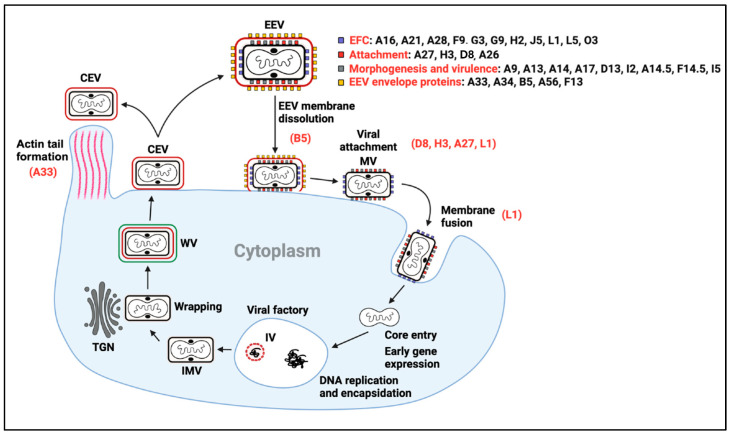
Schematic representation of the replication cycle of Orthopoxviruses. Above, a schematic of an enveloped particle (EV). The outer membrane is represented by a red line, and the inner by a black line. The surface proteins of each membrane are indicated alongside. Below, the main steps of viral replication are indicated. The main neutralization determinants identified so far are highlighted in red, alongside the specific viral step they are involved in. Key abbreviations include MV for mature virion, IV for immature virion, IMV for intracellular mature virion, WV for wrapped virion, CEV for cell-associated virion, EEV for extracellular enveloped virion, EFC for entry fusion complex, and TGN for trans-Golgi network. With permission from Pablo Guardado-Calvo [37].

VACV has been used as a model system to study poxviruses. There are two forms of infectious virions, each with different receptor-binding proteins and surface proteins. They are the extracellular enveloped virus (EEV), responsible for spreading within the body, and the intracellular mature virus (IMV), released on cell lysis and primarily responsible for transmission to other hosts. The IMV enters cells via endocytosis at low pH or fusion with the plasma membrane at neutral pH. This is followed by depositing the vial core into the cytoplasm [35]. The IMV has an entry fusion complex (EFC) of 11 proteins, namely A16, A21, A 28, F9, G3, H2, J5, L1, L5, and O3. This is necessary for the virus to enter cells [36]. There are four IMV attachment proteins, namely A27, H3, D8, and A26 [35,37]. In Figure 1, the surface proteins of VACV are given, which are targets for IMV and EEV neutralization, as well as those in the EFC of IMV. A33, A35, A56, F13, and B5 are components of the EEV envelope [38]. Cell-associated virus (CEV) remains attached at the cell surface and can be propelled into adjacent cells by the actin tails below the membrane surface [39]. An effective poxvirus vaccine must protect from both the IMV and EEV forms.

## 3. Smallpox Vaccines That Protect from Mpox

**Original Smallpox Vaccines:** The early smallpox vaccines came from several sources, including cowpox virus, VACV, and horsepox virus [40]. The vaccines used to eradicate smallpox were a mixture of VACV strains grown on live sheep or cattle. These vaccines could complete the viral replication cycle in people and be transmitted from the vaccinated individuals to others. Dryvax^®^ was one of the most widely used vaccines during the WHO smallpox eradication campaign [41]. More recently, ACAM2000 was derived from Dryvax^®^ [42]. This was manufactured using modern cell-culture methods derived from a single VACV plaque. ACAM 2000 is also a replicating VACV vaccine that can be transmitted to persons who have close contact with the vaccinated person. These vaccines can cause serious adverse events, including death, in HIV-positive people and others with congenital or acquired immunodeficiency disorders. In areas such as South Africa, with high HIV prevalence, this is seen as a significant problem. Contraindications for vaccination with live replicating VACV-based vaccines are pregnancy, immunosuppression, and a history of cardiac complications [43]. The reported rates for any cardiovascular event in vaccinated people in the US military were 113.5/100,000 for the ACAM2000 vaccine and 439.3/100,000 for the Dryvax vaccine. Neurological event rates were 2.12/100,000 and 1.11/100,000 for ACAM2000 [44]. Nevertheless, the WHO recommends ACAM2000 if no other vaccines are available.

**Safer Smallpox Vaccines:** Smallpox eradication meant routine vaccination was no longer necessary to prevent this disease. However, the threat of using this virus as a biological weapon, or alternatively an unforeseen disease outbreak, resulted in many countries stockpiling smallpox vaccines [43]. While there are large stockpiles of vaccines such as Dryvax and ACAM 2000, there are also next-generation vaccines with a better safety profile. The next-generation vaccine, called Jynneos in the USA and Imvanex in Europe, is based on an attenuated or weakened form of VACV called modified vaccinia Ankara (MVA) from the Bavarian Nordic (BN) in Denmark. MVA was attenuated by serial passage through chick embryo fibroblasts and has six large genomic deletions compared with VACV [45]. MVA-BN cannot complete its replication cycle in humans and is safe in immunosuppressed individuals [46]. However, there is still a higher risk of adverse cardiovascular events after two MVA-BN immunizations (relative risk [RR] 4.07, 95% confidence interval [CI] 1.10–15.10) [47]. No increased risk of adverse events was observed after one immunization; however, two immunizations are needed to protect against mpox.

In the recent mpox outbreaks in the USA and Great Britain, the estimated efficacy of two MVA-BN vaccinations was 80%, with no hospitalizations of vaccinated people, and one immunization was about 60% effective in preventing clinical manifestations among both HIV-positive and HIV-negative persons [48,49]. However, the antibodies wane with time and are not detectable one year after immunization. The duration of protection is unknown, but a non-replicating vaccine is expected to give less protection than the replicating vaccines. Evidence shows that waning immunity makes people susceptible to MPXV infection [50,51]. The vaccine cost is also an obstacle as Bavarian Nordic sold their vaccine at USD 110 a dose. The MVA-BN vaccine has been approved for use in adults, but trials are still being conducted to register it for use in children. Another recently available vaccine is LC16m8 made by KM Biologics in Japan. While LC16m8 contains replicating VACV, it is a more attenuated vaccine than the original VACV-based vaccines. LC16m8 has all the open reading frames of VACVs except for the disrupted EEV envelope gene B5R [52]. Therefore, it has a better safety profile than ACAM2000. It is also approved for use in children. LC16m8 requires one immunization, while MVA-BN-based vaccines require two immunizations [53]. WHO recommends both the MVA-BN and LC16m8 vaccines.

## 4. Correlates of Protection for Poxvirus Infections

The risk of bioterrorism with the virus causing smallpox (Variola Virus) has stimulated vaccine research on VAVC and MPXV as model organisms. Initial experiments in mice testing smallpox vaccines demonstrated that antibodies were essential to protect from VACV disease. CD4+ nor CD8+ T cells alone were not sufficient to protect from disease. Still, they played a role in controlling sublethal infections in mice that had not been immunized [54]. In an MPXV challenge model in macaques after VACV vaccination, VACV-specific B-cell responses were essential for protection—passive transfer of neutralizing VAVC antibodies protected from severe disease [55]. In line with this result, intravenous vaccinia immunoglobulin is available for passive immunization for people exposed to virulent Orthopoxviruses such as MPXV [56].

## 5. Research on Next-Generation Poxvirus Vaccines

The present poxvirus vaccines are based on attenuated viruses. These vaccines are generally heat-stable when freeze-dried and relatively inexpensive to produce. However, there is also a justification for “next-generation” vaccines, so poxvirus vaccines not based on live attenuated viruses are being developed. Vaccines based on subunit vaccines produced in cell culture, bacterial production systems, or plants are being investigated. There is some research on DNA- or RNA-vectored vaccines or viral-vectored vaccines. The vaccines generally target both the EEV and the IMV. Different platforms have different strengths. The proteins selected as vaccine targets to induce neutralization should be in the correct conformation to induce these responses. Since they are mostly glycosylated proteins [37], it is unlikely they will be correctly produced in bacteria [57]. Glycosylation can be modified in plants to mimic the original conformation [58] while nucleic acid-based vaccines could be expected to produce the proteins in the correct conformation.

Poxviruses make good vaccine vectors, and it is of note that MVA-BN expressing Ebola virus proteins is now available as part of an Ebola virus vaccine schedule [59]. MVA has also been used as an HIV vaccine vector in clinical trials [60,61,62,63]. Vaccination campaigns against mpox tend to be targeted, and it is unlikely the entire population will be vaccinated [64,65]. A dual vaccine that protects against mpox and other important diseases such as respiratory syncytial virus [66] for influenza virus [67] could significantly broaden the target populations. Several such vaccines are in development based on MVA and LC16m8 [66,67].

Although not yet commercialized, LSDV has been used to develop a number of dual vaccines for cattle against LSD and other pathogens, including rabies [68], Rift Valley fever virus [69], bovine ephemeral fever [70], and East Coast fever [71,72].

## 6. Mpox Vaccine Development

There is a need for better MPXV vaccines that induce long-lasting antibodies and protection, as well as for cheaper vaccines. Natural infection with MPXV-induced antibodies is at a significantly higher titer than the MVA-BN vaccine, while the T cell responses were comparable [73]. There are reports of incomplete protection in people vaccinated with MVA-BN [48,74]. The correlate of protection for mpox vaccines is still to be defined, but there is a trend for antibodies to be correlated with protection [75]. In a non-human primate model testing an mRNA vaccine, a range of antibody functions were shown to be important in controlling MPXV viraemia, including neutralizing and Fc-effector functions against both the intracellular and extracellular forms of the virus (IMV and EEV). Determinants of lesion control by specific antibodies include opsonophagocytic activity and neutrophil/natural killer (NK) cell-targeted functions [76].

More immunogenic vaccines are needed to protect against mpox. While the MPXV has approximately 190 genes, selected genes are targeted for recombinant technology to construct subunit vaccines. There are examples of various combinations of genes that have been successful in candidate mpox vaccines, as shown in the Table 1. MPXV A29, A35, B6, and M1 are next-generation vaccines’ most commonly chosen proteins. Poxviruses enter cells by fusion between the viral and host membranes [36]. MPXV M1 and A29 proteins are involved in cellular entry of the IMV, and the A35 and B6 proteins are involved in the spread of EEV [76]. mRNA vaccines made with modified versions of the proteins, B6, A35, and M1 with engineered transmembrane regions and cytoplasmic tails that localized to cell surfaces, and A29 with an influenza virus H1 HA signal peptide that was secreted were expressed well in transfected cells. They elicited superior neutralizing Ab titers in mice compared to MVA [77]. Another approach to improve the current poxvirus vaccines is by adding MPXV antigens [78].

**Table 1:** Summary of Some Key Papers on Next-Generation Mpox Vaccines.

Mpox Vaccine Platform	Intracellular Mature Virion (IMV) Protein Targets	Extracellular Enveloped Virion (EEV) Protein Targets	Challenge Result	Ref
From Vazyme, Nanjing, China (platform not given) adjuvant was QS-21	MPXV A29L, M1R—soluble form with His tag	MPXV A35R, B6R soluble form with His tag	Protects mice from MPXV challenge.	[79]
Baculovirus expressionAdjuvant aluminum hydroxide (alum) ± CpG	VACV L1, A27	VACV B5, A33	Alum adjuvanted vaccines provided only partial protection, but the addition of CpG provided full protection from lethal intravenous MPXV challenge in macaques.	[80]
DNA vaccine prime-boost with the *E. coli*-produced proteins	VACV L1R, A27L	VACV L1R, B5R	DNA vaccine or proteins did not protect Rhesus macaques from MPXV challenge when given alone. Prime-boost with DNA-protein gave better protection and resulted in mild disease.	[81]
Expi293F expression systemAlum adjuvant	Structural-based design resulted in a two-in-one immunogen with MPXV A35 bivalently fused with M1 (called DAM).		Induced much higher antibody titres than the VACV and protected from lethal VACV challenge in mice.	[82]
mRNA	MPXV M1 H3	MPXV A35, B6	Prevented death and suppressed lesions after lethal clade I MPXV challenge in cynomolgus macaques.	[83]
mRNA	MPXV A29, M1	MPXV B6, and A35	Complete survival in mice against lethal VACV challenge with mRNA vaccine and provided superior protection against weight loss compared with MVA.	[77]
mRNA-1769	MPXV A29, M1	MPXV B6, and A35	MVA and mRNA-1769 conferred complete protection from lethal MPXV challenge in cynomolgus macaques. There were 10-fold fewer lesions and reduced duration of disease lower viremia in the mRNA cohort compared to MVA.	[76]

Note: MPXV A29, A35, B6, and M1, are orthologs of VACV A27, A33, B5, and L1.

## 7. Capripoxvirus Vaccines

There are three species in the Capripoxvirus genus—lumpy skin disease virus (LSDV), which causes disease in cattle; sheeppox virus (SPPV); and goatpox virus (GTPV), which causes sheeppox and goatpox, respectively. Present vaccines are generally live attenuated viruses, and there is some cross-protection between the three different species. The original live attenuated LSDVs originated from the Neethling field strain of LSDV, which is marketed under various names around the world. In India, an attenuated vaccine was produced after passaging fifty times in Vero cells. The vaccine protects from LSDV challenge and is now being commercialized for use in Asia [84]. Due to the cross-reactivity between members of the Capripoxvirus genus, live attenuated GTPV and SPPV are also used to vaccinate cattle against LSDV [57]. GTPV-based vaccines have been reported to show the same protection against LSD as LSDV-based vaccines [58,59]. However, this is not the case with SPPV vaccines [72]. Other initiatives to improve live LSD vaccines include targeted approaches to deletion of virulence genes. LSDV knock-outs of either an interleukin10-like (LSDV005) or interferon-γ receptor-like genes (LSDV008) have been made, but they did not reduce virulence enough to be used as a vaccine [85]. Four genes were deleted to attenuate the virulent LSDV Atyrau/KZ. These included LSDV008 coding for an interferon-γ receptor-like protein; LSDV142, which encodes a protein of the Bcl-2-like protein family and inhibits both apoptosis and activation of the pro-inflammatory transcription factor nuclear factor kappa B; and LSDV066, a gene which encodes thymidine kinase. The attenuated vaccine was safe and protected from virulent LSDV challenge [86].

In an attempt to improve LSDV vaccine, a synthetic stabilized superoxide dismutase (SOD) gene was introduced to stabilize this site in the virus. In addition, SOD was deleted in another recombinant. In recombinants, LSDV with these modifications both protected against LSDV challenge. The SOD knock-out virus gave a weaker immune response to LSDV and the recombinant proteins [70,87].

As LSDV spreads, there is a need for vaccines that could be used in countries where LSDV is not endemic. If a country has had to vaccinate against LSD, presently there is no test to differentiate infected from vaccinated animals (DIVA). For the country to be declared free of disease, DIVA is essential [88]. This is not yet possible on the attenuated vaccines but should be possible with the next-generation vaccines. There is also hesitancy to use live attenuated vaccine after the recombination events reported in Asia [72] leading to several initiatives, including killed/inactivated vaccines [89].

The next-generation Capripoxvirus vaccines are generally in early-stage development, and most have not been tested in challenge experiments in their target host. Early work on subunit vaccines was conducted at the Pirbright laboratory, where KS-1 P32 was produced in *Escherichia coli* as a glutathione -S- transferase fusion. This protein was tested in goats and gave partial protection from GTPV challenge [90]. In line with the strategy used for VACV, two GTPV vaccines based on the A27, L1, A33, and B5 VACV homologues were made using Semliki Forest virus replicon-based DNA vaccines. In goats, these vaccines provided partial protection from GTPV challenge with reduced disease symptoms. They also reduced the symptoms induced by the existing GTPV vaccine [91]. Both the Pirbright subunit vaccines and Semliki Forest virus replicon-based DNA vaccines appeared to protect against disease rather than transmission, which raises the question of the titer of neutralizing antibodies needed for protection. Were T cells responsible for protection from severe disease?

Different production platforms, including plant-based platforms, are being investigated. Plants are considered a good platform for vaccine production as they are a cost-effective manufacturing platform [92]. Plant-based subunit Capripoxvirus proteins have been produced in *Nicotiana tabacum*. SPPV 117 was produced in chloroplasts and was shown to react with sheep serum [93]. The SPPV L1R hydrophilic domain has been made in transplastomic tobacco plants [94]. The LSDV orthologs of VACV A27L and L1R were produced in *E. coli* and formulated with Montanide Gel 01PR adjuvant. These vaccines were tested in rabbits, and neutralizing antibodies were detected after the second immunization [95]. Dissolvable microneedle patches have been tested in mice to deliver the LSDV orthologs of VACV L1, A27, A33, and B5 synthesized in *E. coli*. The patches were stable for 4 months at room temperature, an advantage over most subunit vaccines’ storage requirements. Both neutralizing and cellular immune responses were detected [96].

While most of the novel LSD vaccines are based on proteins selected as orthologs of VACV proteins, some designs select epitopes using various immunoinformatic approaches [97,98,99]. They have not yet been tested in cattle.

## 8. Conclusions

LSD and mpox are largely diseases found in less advantaged parts of the world, and so some emphasis on cost-effective vaccines is important. While the recent outbreaks of mpox are in countries that can afford to purchase vaccines, the ongoing outbreaks in Africa are not in the same category. If mpox vaccination had been implemented in Africa, the present outbreaks within and outside of Africa could have been prevented. It is a problem that Africa does not manufacture the majority of their vaccines, and recently efforts have been made to improve this situation [100,101]. The success of mRNA vaccines for COVID-19 has stimulated the use of this technology for next-generation vaccines. Other approaches are also being studied, including DNA-based and replicating nucleic acid vaccines. Similar approaches to vaccine design can be applied to both MPXV and LSDV, with nucleic acid-based vaccines being the most flexible approach. mRNA-based mpox vaccines have been shown to be successful in macaque challenge experiments [76,83], so it is likely that these vaccines will be commercialized. While mRNA vaccines are not yet available for cattle, this approach will likely be feasible [88]. However, even if successful, unless mRNA vaccine production can be made less expensive so that developing country economies can afford to purchase them, other manufacturing platforms still need to be developed. Proteins produced using different cost-efficient production platforms must be tested in the appropriate challenge models to ensure protection from disease. Another promising approach is making recombinant vaccines that protect from the mpox disease and other critical diseases so that the general population can be vaccinated and protected from both diseases. Dual vaccines against LSD and other pathogens are also an appealing way of immunizing cattle against multiple diseases and making vaccination more cost-efficient.
